# Mad River Valley Dental Association

**Published:** 1860-05

**Authors:** 


### MAD RIVER VALLEY DENTAL ASSOCIATION.

This Association held its fourth quarterly meeting at the office of Dr. B. A. Rose, in Urbana, on Thursday, April 5th.

The meeting was called to order at 2 o'clock, by the President, Dr. G. WATT.

The minutes of the last meeting were read by the Secretary, Dr. G. L. PAINE, and approved.

The following Dentists were elected members of the Association, viz: Drs. W. A. Pease and C. Bradley, of Dayton; Drs. D. W. Harris and N. W. Williams, of West Liberty.

The members of the Association entered into a discussion of Mechanical Dentistry, including the taking of casts and swedging of plates. Each member present gave his own experience in his practice in these operations, and the exchange of opinions on the *modus operandi* of each was interesting and valuable to the profession. After a full discussion on this subject, the meeting adjourned to meet at 7 o'clock.

In the evening Dr. Clippinger read a very entertaining essay upon "Taking casts of the mouth in plaster Paris." The essay was ordered to be published in the *Dental Register of the West.*

The subject for discussion in the evening was "Filling Teeth." Much interest was elicited on this subject, and a prolonged discussion was had by the members. Much information of value was received through the experience of each member.

The following gentlemen were elected delegates to the National Society, which meets in July, at Washington, D. C., viz: W. A. Pease, A. A. Blount, and G. L. Paine.

The following gentlemen were appointed to read Essays upon subjects relating to Dentistry, at the next regular meeting:

Dr. A. A. Blount, of Springfield; Drs. E. M. Lee, J. G. Palmer, and B. A. Rose, of Urbana.

At half-past nine o'clock the Association adjourned to meet at the office of Dr. W. A. Pease, in Dayton, July 3d, 1860.

After the adjournment, the Dentists consumed some time in conversation upon Mechanical and Operative Dentistry.

The following is a list of members present:

A. A. Blount, Springfield; W. A. Pease, C. Bradley, Dayton; G. Watt, G. L. Paine, Xenia; D. W. Harris, E. W. Williams, West Liberty; S. Clippinger, Bellefontaine; J. H. Paine, Germantown; E. M. Lee, J. G. Palmer, B. A. Rose, Urbana.

